# Genetic Diversity of the Coat Protein of Olive Mild Mosaic Virus (OMMV) and Tobacco Necrosis Virus D (TNV-D) Isolates and Its Structural Implications

**DOI:** 10.1371/journal.pone.0110941

**Published:** 2014-10-28

**Authors:** Carla M. R. Varanda, Marco Machado, Paulo Martel, Gustavo Nolasco, Maria I. E. Clara, Maria R. Félix

**Affiliations:** 1 Laboratório de Virologia Vegetal, Instituto de Ciências Agrárias e Ambientais Mediterrânicas Universidade de Évora, Évora, Portugal; 2 Departamento de Ciências Biológicas e Bioengenharia, Faculdade de Ciências e Tecnologia da Universidade do Algarve, Faro, Portugal; 3 Laboratório de Virologia Vegetal, Universidade do Algarve, Faro, Portugal; Washington State University, United States of America

## Abstract

The genetic variability among 13 isolates of *Olive mild mosaic virus* (OMMV) and of 11 isolates of *Tobacco necrosis virus D* (TNV-D) recovered from *Olea europaea* L. samples from various sites in Portugal, was assessed through the analysis of the coat protein (CP) gene sequences. This gene was amplified through reverse transcriptase polymerase chain reaction (RT-PCR), cloned, and 5 clone sequences of each virus isolate, were analysed and compared, including sequences from OMMV and TNV-D isolates originally recovered from different hosts and countries and available in the GenBank, totalling 131 sequences. The encoded CP sequences consisted of 269 amino acids (aa) in OMMV and 268 in TNV-D. Comparison of the CP genomic and amino acid sequences of the isolates showed a very low variability among OMMV isolates, 0.005 and 0.007, respectively, as well as among TNV-D isolates, 0.006 and 0.008. The maximum nucleotide distances of OMMV and TNV-D sequences within isolates were also low, 0.013 and 0.031, respectively, and close to that found between isolates, 0.018 and 0.034, respectively. In some cases, less variability was found in clone sequences between isolates than in clone sequences within isolates, as also shown through phylogenetic analysis. CP aa sequence identities among OMMV and TNV-D isolates ranged from 84.3% to 85.8%. Comparison between the CP genomic sequences of the two viruses, showed a relatively low variability, 0.199, and a maximum nucleotide distance between isolates of 0.411. Analysis of comparative models of OMMV and TNV-D CPs, showed that naturally occurring substitutions in their respective sequences do not seem to cause significant alterations in the virion structure. This is consistent with a high selective pressure to preserve the structure of viral capsid proteins.

## Introduction

*Olive mild mosaic virus* (OMMV) and *Tobacco necrosis virus D* (TNV-D) originally placed in the Necrovirus genus, were recently divided and included into the new genera *Alphanecrovirus* and *Betanecrovirus*, respectively, based on the level of sequence diversity in their polymerases.

Complete genomic sequences of TNV-D were obtained from isolates recovered from bean in England [1], tobacco in Hungary [2] and, more recently, from an olive tree in Portugal [3].

In 2004, an olive isolate initially identified as a TNV-D isolate based on the sequencing of the coat protein (CP) gene [4] was later considered a distinct species, OMMV [5], following the complete sequencing of its genome. Based on the deduced amino acid (aa) CP sequence, OMMV showed 85.1% identity with that of TNV-D [3], which explains its initial diagnosis as a TNV-D isolate. Since then, OMMV has been recorded infecting other hosts such as spinach in Greece [6] and tulip in the Netherlands, revealing that OMMV is part of the viral complex associated with the Augusta disease, previously ascribed to TNV [7].

Differentiation between OMMV and TNV-D is only possible through PCR based assays using specific primers [8] or through complete genome sequencing. Thus, TNV isolates referred in earlier literature may reveal themselves to be either TNV-D or OMMV.

OMMV and TNV-D particles are oligomers consisting of 180 copies of the CP polypeptide [5]. Both viruses have isometric particles, *ca*. 28 nm in diameter, with single-stranded positive-sense RNA and a genome of *ca.* 3.7 kb in length. OMMV genome has 5 Open Reading Frames (ORF) and TNV-D has 6. The 5′–proximal ORF1 of OMMV encodes a polypeptide of 202 aa with a molecular weight (MW) of 23 kDa (p23) and that of TNV-D has 22 KDa (p22) MW. ORF1RT results from the read-through of the amber stop codon, and encodes a 82 kDa protein predicted to be the viral RdRp. OMMV ORF2 overlaps ORF1RT by 17 nts and encodes a 8 kDa polypeptide with 73 aa (p8) and ORF 3 encodes a 56 aa polypeptide with a molecular mass of 6 kDa (p6). These two small proteins are predicted to be involved in virus movement based on the high aa sequence identity with the OLV-1 movement proteins p6 and p8. As for TNV-D, ORFs 2, 3 and 4 are predicted to encode small peptides with about 7 kDa designated p7_1_ (62 aa), p7a (65 aa) and p7b (66 aa) respectively. The 3′–proximal ORF5 of OMMV and TNV-D encode a 269 aa polypeptide with 29 kDa (p29), identified as the virus CP.

Most CPs of plant icosahedral positive-stranded RNA viruses have four distinct structural domains: an ‘R’ domain involved in the interaction with RNA, a connecting arm ‘a’, a central shell domain ‘S’ and a C-terminal projecting ‘P’ domain. Necroviruses particles do not have a protruding domain [9], [10]. The ‘S’ domain comprises 8 anti-parallel beta-strands, which form a twisted sheet or jelly-roll fold [11] and presents a signature pattern, consisting of 26 amino acid residues ([FYW]-x-[PSTA]-x(7)-G-x-[LIVM]-x-[LIVM]-x-[FYWIL]-x(2)-D-x(5)-P). In OMMV this pattern was detected in positions OMMV CP 134 to 159 aa [4], [12]. OMMV and TNV-D CP show significant homology (≈ 45% identity) with the *Tobacco necrosis virus A* coat protein, whose quaternary structure was solved at 2.25 Å resolution [13]. The quaternary structure of the OMMV coat arrangement has been determined based on the TNV-A structure through comparative modelling approaches and the effect of two mutations in the virion structure was assessed [14].

The CP has been shown to be involved in many non-structural functions such as virus movement within the plant, genome activation and elicitation of symptoms, as well as in suppression of RNA silencing and vector transmission [15]–[17]. Recent studies showed that a single mutation in the CP gene of OMMV was responsible for the loss of transmission by the fungus *Olpidium brassicae* [14].

RNA viruses are expected to undergo high mutation rates potentially leading to a substantial genetic variation. Continuous generation of new mutants gives rise to a high diversity in nucleotide sequences among viruses leading to populations made up of many different yet related genomes. Conversely, as a result of factors such as natural selection, bottleneck effect occurrences and even natural host range, this genetic diversity has often showed to be lower than expected [18]–[21]. Recently, analysis of the genetic diversity of the CP of the alphanecrovirus *Olive latent virus* 1 (OLV-1), that shares an identity of 49.6% with OMMV CP and 48.8% with that of TNV-D [3], [5], showed a very low value of genetic diversity, 0.02, among 25 isolates obtained from different hosts and locations [22].

Knowledge on the diversity in the viral CP gene will help to understand how viruses become adapted to hosts and vectors and contribute to more efficient and durable diagnostic methods. In this work we analysed the CP molecular diversity of the two viruses, OMMV and TNV-D, that share an economically important host, olive, and determined the implications of that diversity in the virions structure.

## Materials and Methods

Viral isolates used in this study were obtained from olive fruits and leaves collected during the year of 1995, during two research projects of University of Évora (1995–1998 - Project PAMAF-IED N° 4057 ‘Valorization of Olea europaea L. cultivars “Negrinha de Freixo” and “Santulhana” in Trás-os-Montes region of Portugal’, coordinated by Estação Agronómica Nacional, Oeiras. Person in charge: Eng° Fausto António Leitão; and 1995–1998 - Project PAMAF-IED N° 2064 ‘Selection of clones of Olea europaea L. cultivars used in the production of Moura olive oil for the renovation of olive orchards in Serpa and Moura, south of Portugal’, coordinated by Instituto de Biologia Experimental e Tecnológica, IBET, Oeiras. Person in charge: Professor Pedro Fevereiro. No protected species were sampled. Isolates have been maintained, since then, in *Chenopodium murale* plants in Laboratory of Plant Virology of University of Évora, and have been used in several studies.

### Virus isolates

Thirteen OMMV isolates and eleven TNV-D isolates obtained from field olive trees growing in different regions of Portugal were used in this study. Viral isolates were collected during the year of 1995, during two research projects of University of Évora. Isolates have been maintained, since then, in *Chenopodium murale* plants in Laboratory of Plant Virology of University of Évora, and have been used in several studies.

Some of the field isolates revealed to be either OMMV (five) or TNV-D (three) whereas eight were a complex of both (Table 1). OMMV3 resulted from the inoculation in *C. murale* plants of OMMVpUFLOMMV-3 (gb|HQ651832.1|), a full length clone derived from GP isolate (NC_006939.1); OMMVL11 is an OMMV3 variant obtained after 15 serial passages of single local lesions in *C. murale* plants which has shown *O. brassicae* non transmissible [17]. V8ia1 isolate was obtained following 10 serial passages of an initial single local lesion induced in *C. murale* plants by V8i.

**Table 1:** OMMV and TNV-D isolates present in single and in double infected plant samples and Genbank sequences used in coat protein variability analysis.

Plant Host and Virus origin	Virus	Viral Isolates	Accession number*
			
Olive, Portugal	OMMV	A4P5	KM355270 (1); KM355271 (2); KM355272 (2)
		V4	KM355275 (5)
		V8	KM355276 (4); KM355277 (1)
		OMMV3	KM355247 (3); KM355248 (2)
		OMMVL11	KM355249 (5)
		V8i _OMMV	OMMV: KM355278 (4); KM355279 (1)
		V10_OMMV	OMMV: KM355280 (1); KM355281 (1); KM355282 (1); KM355283 (2)
		gp _OMMV	OMMV: KM355250 (1); KM355251 (2); KM355252 (2)
		A1P2_OMMV	OMMV: KM355253 (1); KM355254 (1); KM355255 (1); KM355256 (1); KM355257 (1)
		A4P2_OMMV	OMMV: KM355258 (1); KM355259 (2); KM355260 (1); KM355261 (1)
		A5P2_OMMV	OMMV: KM355262 (2); KM355263 (1); KM355264 (1); KM355265 (1)
		A10P2_OMMV	OMMV: KM355266 (2); KM355267 (1); KM355268 (1); KM355269 (1)
		A6P5_OMMV	OMMV: KM355273 (2); KM355274 (3)
	TNV-D	gp_TNV-D	TNV-D: KM355307 (2); KM355308 (3)
		V4PB	KM355284; KM355285; KM355286; KM3555287; KM355288
		V6	KM355309 (1); KM355310 (1); KM355311 (1); KM355312 (2)
		V8ia1	KM355294 (1); KM355295 (2); KM355296 (1); KM355297 (1)
		V8i_TNV-D	TNV-D: KM355313 (5)
		V10_TNV-D	TNV-D: KM355289 (2); KM355290 (1); KM355291 (1); KM355292 (1)
		A1P2_TNV-D	TNV-D: KM355298 (2); KM355299 (2); KM355300 (1)
		A4P2_TNV-D	TNV-D: KM355301 (2); KM355302 (3)
		A5P2_TNV-D	TNV-D: KM355293 (5)
		A10P2_TNV-D	TNV-D: KM355305 (3); KM355306 (2)
		A6P5_TNV-D	TNV-D: KM355303 (4); KM355304 (1)
			GenBank sequences
Olive, Portugal	OMMV	GP	NC_006939.1
		GP puFLOMMV3	HQ651832.1
		GP puFLOMMV4	HQ651833.1
		GP puFLOMMV5	HQ651834.1
Tulip, The Netherlands		TNV5	EF201607.1
		Inzell	EF201606.1
		BKD	EF201605.1
Spinach, Greece		OMMV-spinach	JQ288895.1
Tobacco, Hungary	TNV-D	Hungarian	NC_003487.1
Bean, United Kingdom		English	D00942.1
Olive, Portugal		Portuguese	FJ666328.1

* Number of clones that present the same sequence are between brackets.

Additional CP gene sequences from 3 isolates of TNV-D and of 8 OMMV of various origins were retrieved from Genbank and included in the present study (Table 1).

### Nucleic acid extraction

Total RNA was extracted from 100 mg of symptomatic leaves of *C. murale* plants previously inoculated with the isolates indicated in Table 1, following maceration in liquid nitrogen and use of the commercial RNeasy Plant Mini Kit (Qiagen) according to manufacturer's instructions.

### RT-PCR

For cDNA synthesis, 1 µg of total RNA was used together with 1.5 µg of random hexamers (Promega) followed by denaturation at 70°C for 10 minutes and incubation on ice for 15 minutes. Reverse transcription (RT) reaction was performed in the presence of 200 U of M-MLV reverse transcriptase (Invitrogen), 50 mM Tris-HCl pH 8.3, 75 mM KCl, 3 mM MgCl_2_, 10 mM DTT and 0.5 mM dNTPs.

For amplifications, initially, one common pair of primers was designed based on OMMV and TNV-D Genbank sequences, to amplify their CP ORFs (OMMVTNVDcoat5′: TAATCATGCCTAAGAGAGG and OMMVTNVDcoat3′: ATCCTTCCATTAACGTTTA) and used. Later, two pairs of specific primers, one designed to amplify the CP ORF of OMMV (OMMVcoat5′: GACATTTCGCAACTCTCT and OMMVcoat3′: CACAACGATGGGTGAGTTGC) and the other designed to amplify the CP ORF of TNV-D (TNVDcoat5′: TCGGAGGATCAACCACTACAAA and TNVDcoat3′: CCGGAAGACGGGTCTATGAAA) were used. In all reactions, one μL of cDNA was used in a 50 µL reaction with 2.5 U of FideliTaqDNA Polymerase (USB corporation) performed in 10 mM Tris HCl (pH 8.6), 50 mM KCl, 1.5 mM MgCl2, 0.2 mM dNTPs, 0.3 µM of each primer. Amplifications were performed in a Thermal Cycler (BioRad) following initial denaturation at 94°C for 1 min, 35 cycles at 94°C for 1 min, 52°C for 2 min and 68°C for 1 min and 30 seconds, and a final extension step of 68°C for 5 min. The use of the common primers OMMVTNVDcoat in RT-PCR assays originated a fragment of ≈813 bp for OMMV and ≈810 bp for TNV-D and the use of the specific pair of primers for OMMV and TNVD, originated a fragment of ≈877 bp and ≈872 bp, respectively.

### Cloning and sequence analysis

RT-PCR products were purified using GFX PCR DNA Purification kit (GE Healthcare Biosciences) and cloned into pGEM-T easy vector (Promega), in accordance with the manufacturer's instructions. Plasmid DNA was extracted from *E.coli* JM109 cells using GenElute HP Plasmid Miniprep kit (Sigma) in accordance with the manufacturer's instructions, after growing cells in low salt LB medium (1% tryptone, 0.5% yeast extract, 0.5% NaCl, pH 7.5) supplemented with 100 µg/mL of ampicillin and grown overnight at 37°C at 175 rpm. DNA sequencing reactions were performed on both strands, by Macrogen (The Netherlands). Five complete CP sequences for each isolate were determined. Sequences were deposited in GenBank database. These, as well as those of the OMMV and TNV-D CP gene sequences available from the GenBank database (Table 1), totalling 131 sequences, were compared. The search for homologous sequences was done using BLAST. Multiple sequence alignment was performed with BioEdit 7.1.3.0 [23] and CLUSTAL W in MEGA 5.2 software [24]. The best fit nucleotide substitution model for these data was the Kimura 2-parameter model in the MEGA 5.2 software, showing the lowest Bayesian information criterion (BIC) score. This model was used to estimate nucleotide distance, diversity and phylogenetic relationships which were inferred using neighbour-joining (NJ) method. To validate phylogenetic tree analysis from the NJ method, trees were produced using Minimum Evolution, Maximum Parsimony and Maximum Likelihood methods in the MEGA 5.2 software. Bootstrap analyses with 1000 replicates were performed to evaluate the significance of the inner branches.

Potential recombination events in the aligned sequences were evaluated by RDP, GENECONV, Chimaera, 3Seq and SiSCAN in the RDP4.18 software, using default settings and a Bonferroni-corrected highest *P* value of 0.05. To identify specific amino acid sites under selective constraints, the difference between non synonymous (d_N_) and synonymous (d_S_) substitution rates was estimated for each position in the alignments using the FEL method (0.1 significance level) as implemented in the HYPHY server (http://www.datamonkey.org) [25].

### Secondary structure prediction

An analysis of the potential effect of sequence variants in virion structure was carried using the SIFT algorithm [26], which predicts potentially deleterious mutations based on multiple sequence alignment and position-dependent scoring matrices.

Sequences containing variations marked as potentially deleterious by the SIFT algorithm were set to the JPRED secondary structure prediction server (based on the Jnet neural network algorithm [27]). The predictions for different sequence variants were aligned to highlight differences relative to the reference TNV-D and OMMV sequences.

### Homology Modeling

Given the high similarity of the OMMV and TNV-D sequences (≈85% identity), very similar protein 3D structures were expected. Since the structure of a close homolog (≈45% identity) has been determined by X-ray crystallography (PDB code 1C8N), reliable 3D structural models of the two proteins could be built. To this task, the MODELLER v9 software was used [28]. The models were built as trimmers, since this was the oligomerization state of the asymmetric unit in the 1C8N structure, and the sequences included only the residues present in the crystal structure (78–269 in chains A and B, and 44–269 in chain C). The viral capsid has T3 type icosahedral structure with a total of 180 (60×3) polypeptide chains, and can be produced by replication of the original trimmer using the appropriate symmetry operators. In this study a “minimal” fragment with only 3 trimmers (9 chains) was used, since it contains all possible interfaces between different subunits.

## Results

Amplicons sized ≈810 nt corresponding to the CP gene of OMMV and/or TNV-D were obtained from all isolates when the common primers OMMVTNVDcoat were used in RT-PCR. Each amplicon was cloned and 5 clones per isolate were randomly selected and sequenced. All clone sequences, either from single OMMV or double OMMV and TNV-D infected isolates, revealed a near 100% homology to OMMV in all isolates. Clone sequences obtained from the isolates that were single infected with TNV-D isolates (V4PB, V6 and V8ia1), exhibited near 100% homology to TNV-D.

The use of those primers in amplification reactions applied to RNA obtained from double infected tissues, preferentially amplified OMMV sequences over those of TNV-D. This was overcome using specific primers for each OMMV and TNV-D, resulting in products of the expected size, ≈877 bp and ≈872 bp, respectively.

The CP sequences (5 per isolate) of thirteen OMMV isolates and eleven TNV-D isolates were compared together with the 8 OMMV and 3 TNV-D CP gene sequences available from GenBank, totalling 131 sequences.

All sequences encoding the OMMV CP possessed 269 aa and all sequences encoding the TNV-D CP possessed 268 aa, in accordance to previously published sequences. Pairwise distances of all OMMV sequences ranged from 0.000 to 0.251. The highest value was observed between isolate BKD (from tulip) and most olive isolates, and the lowest, 0.000 to 0.036, was observed among isolates infecting olive, being the highest value (0.036) observed between OMMV clone sequences within sample A1P2. Genetic distance of OMMV clone sequences within samples ranged from very low values, 0.000–0.004 (OMMVL11; V4; OMMV3; A6P5; A5P2; A4P5; V8; V8i; V10; GP), to low, 0.007–0.011 (A10P2; A4P2; A1P2), averaging 0.003, a value near to the OMMV sequence diversity between samples, 0.005, as expected by the coefficient of differentiation (0.607). OMMV sequences from samples OMMVL11 and V4 did not show any diversity.

Pairwise distances of all TNV-D sequences ranged from 0.000 to 0.218. The highest value was observed between the Hungarian and English isolate. As observed for OMMV, the lowest pairwise distance range (0.000 to 0.034) was also obtained among isolates infecting olive. The highest value (0.034) was observed between TNV-D clones of the same sample (A1P2) and between some clones of A1P2 and V8ia1. Genetic distance of TNV-D clone sequences within samples ranged from very low values, 0.000–0.008 (A5P2; V8i; A6P5; A10P2; GP; TNVD; A4P2), to low, 0.012–0.021 (V6; V4PB; V10; A1P2), averaging 0.007, near to the TNV-D sequences diversity between samples (0.006), as shown by the value of the coefficient of differentiation (0.439). TNV-D sequences from samples A5P2 and V8i did not show any diversity.

Pairwise distances of all OMMV and TNV-D sequences taken together ranged from 0.000 to 0.411. The highest value was observed between OMMV and TNV-D clones of sample A1P2 and the lowest was observed between several clone sequences of the same virus from either within or between samples. Diversity among OMMV and TNV-D isolates CP genes reached 0.199.

**Figure 1:**
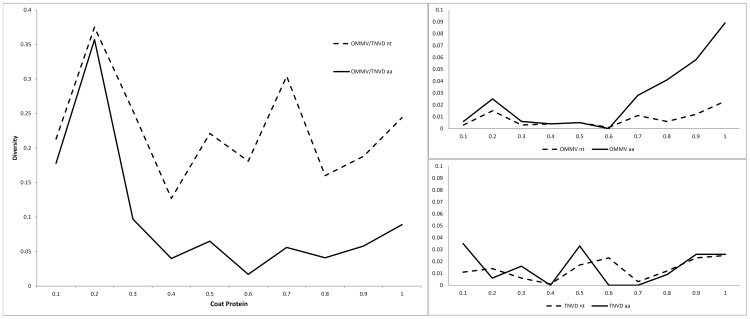
Nucleotide and deduced amino acid diversities along the OMMV and TNV-D CP gene. Left - OMMV and TNV-D aa and nt CP sequences; Right top – OMMV aa and nt CP sequences; Right bottom – TNV-D aa and nt CP sequences. Nucleotide diversity values were obtained in successive windows of 81 nt and amino acid diversity was obtained in successive windows of 26 aa.

Along OMMV CP ORF (Figure 1, top right box), a slight increased variable region is noticed around nt 82 to 163 (aa 28 to 54) (equivalent to 0.2 position on the CP) and from nt 488 to 810 (aa 163 to 269) (0.7 position), more evident in deduced aa sequences. Along the TNV-D CP ORF (Figure 1, bottom right box) the highest diversity was observed in regions nt 1 to 81 (aa 1 to 27) (0.1 position), nt 326 to 406 (aa 109 to 135) (0.5 position) and from nt 650 to 807 (aa 217 to 268) (0.9 position). Comparisons of OMMV and of TNV-D clone CP sequences (Figure 1, left box) have shown the highest diversity (near 0.4) in region nt 82 to 163 (aa 28 to 54) (0.2 position). A high diversity value (0.3) was also observed in region nt 488 to 568 (position 0.7). In terms of amino acid sequence, diversity levels were maintained under 0.06 in region from aa 82 to 243 (position 0.2). From nt 731 to 807 (aa 244 to 268) (0.9 position), there was an increase in nt and aa sequence diversity.

The CP ‘S’ domain starts at aa 53 and ends at aa 265 in OMMV CP (equivalent to positions 0.29 to 0.99 of the CP in figure 1, top right box) and aa 52 to aa 264 in TNV-D CP (equivalent to positions 0.29 to 0.99 of the CP in figure 1, bottom right box) (Jones et al., 2014). The typical signature pattern for this domain ([FYW]-x-[PSTA]-x(7)-G-x-[LIVM]-x-[LIVM]-x-[FYWIL]-x(2)-D-x(5)-P) was recognized at 134 to 159 aa in OMMV and at 131 to 156 aa in TNV-D (equivalent to position 0.6 in figure 1). It consists of highly conserved motif (YIPKCPTTTQGSVVMAIVYDAQDTVP) in all OMMV sequences as well as in all TNV-D sequences except for a single difference in the eighth aa (in bold) where in TNV-D the ‘T’ is substituted by ‘S’. No isolate showed variation in the four amino acids predicted to be involved in Ca^2+^ binding: two residues of aspartic acid (D), one of threonine (T) and one of asparagine (N) [13].

The CP genes of the isolates of both virus species did not show distinct recombination sites, when examined using the RDP software (data not shown) suggesting that recombination has not occurred.

**Figure 2:**
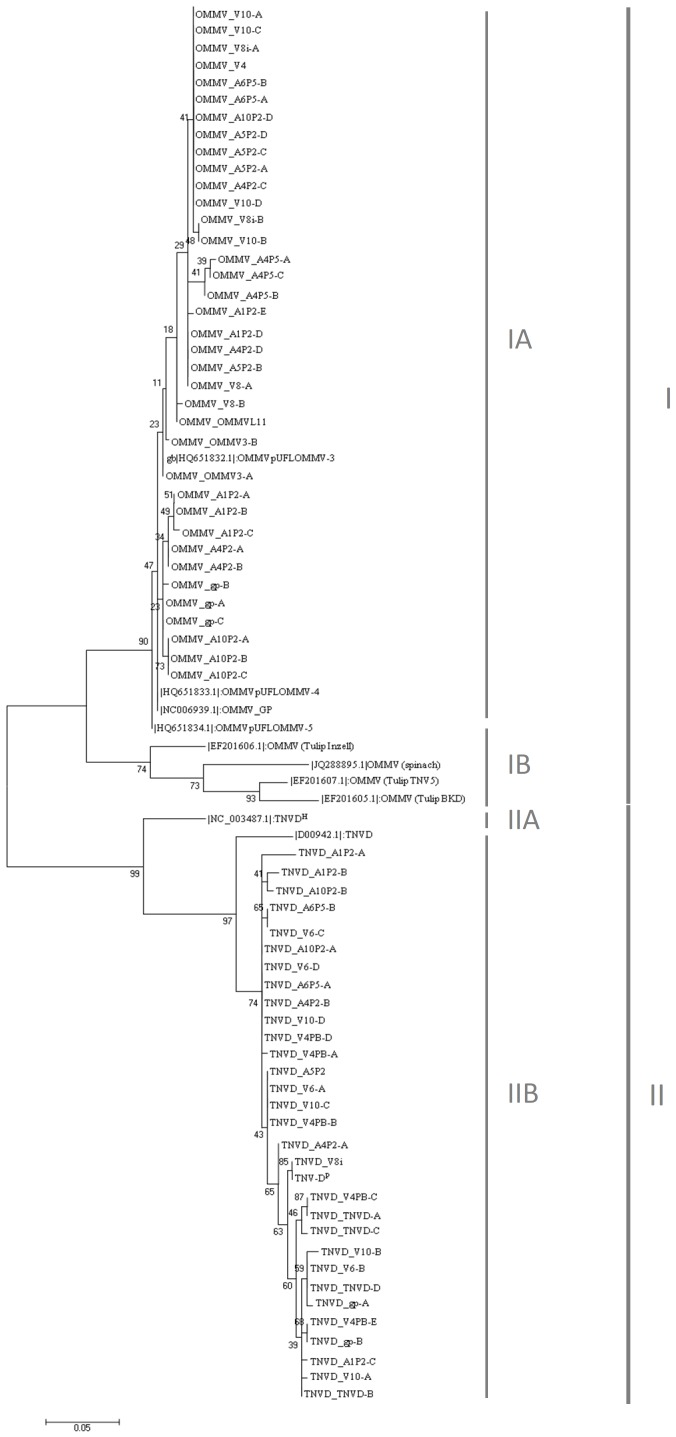
Phylogenetic tree analysis of OMMV and TNV-D isolates based on CP nt sequences. NJ tree was constructed from the sequence alignment of ca. 870 nts of OMMV and TNV-D CP coding region from 65 clones of OMMV and 55 TNV-D isolates and from 11 sequences retrieved from the GenBank database, totalling 131 sequences. Repeated sequences within each isolate were omitted. Each sequence variant was named with a letter (A, B, C,…) following the isolate designation to allow differentiation. Phylogenetic analysis included 78 sequences. Multiple sequence alignments were generated using MEGA 5.1, and phylogenetic tree was constructed by the NJ algorithm, based on calculations from pairwise nt sequence distances for gene nt analysis. Bootstrap analysis was done with 1000 replicates. Numbers above the lines indicate bootstrap scores out of 1000 replicates.

The phylogenetic tree deduced from the CP alignment (Figure 2) revealed segregation of isolates under study into 2 main clusters. As expected from the matrix of sequence identity, isolates were grouped according to the virus species OMMV (I) or TNV-D (II) and each of the 2 main clusters appears divided into 2 subgroups: OMMV olive isolates (I-A) and OMMV tulip and spinach isolates (I-B) and TNV-D tobacco isolate (II-A) and TNV-D bean and olive isolates (II-B). For the phylogenetic tree all clone sequences that were different between them were used instead of using a single consensus sequence per isolate, this way it is visible that clones from different isolates are often closer than clones within an isolate.

The difference between d_N_ and d_S_ at each individual codon was statistically tested by the FEL method to determine if negative selection had some role in the low genetic variability observed. Data showed only 1 positively-selected codon in OMMV (189) and 2 in TNV-D (128, 201), whereas 5 codons were under negative selection in OMMV (44, 172, 230, 257, 264) and 8 codons in TNV-D (136, 155, 187, 199, 238, 261, 263), suggesting negative or purifying selection.

### Secondary structure prediction

**Figure 3:**
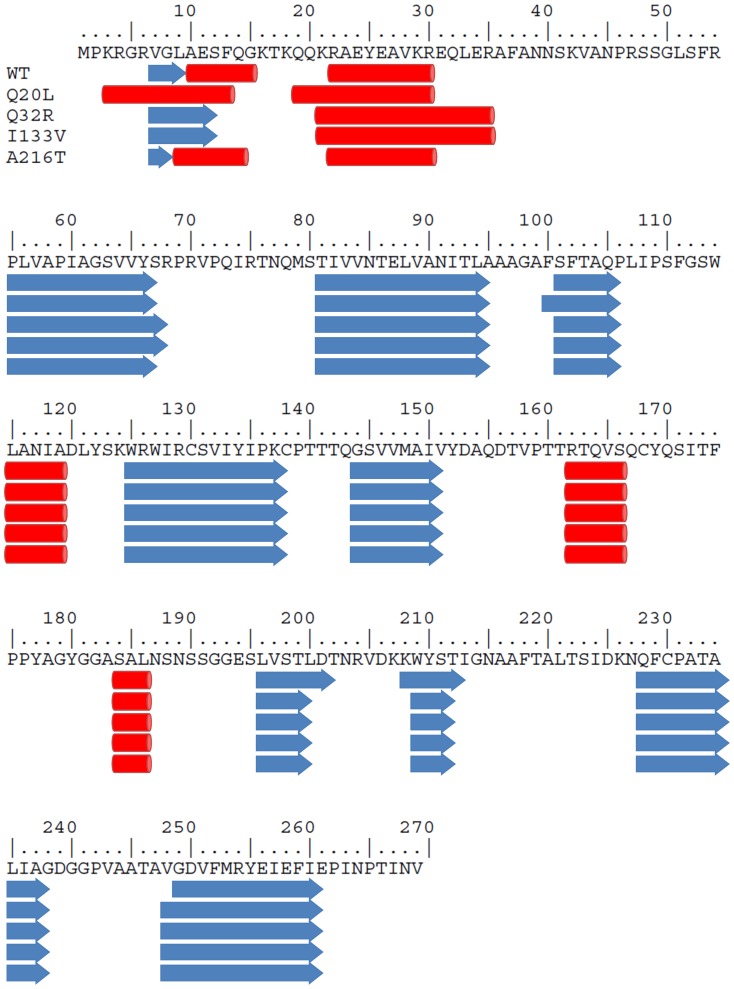
Multiple sequence alignment of the deduced amino acid sequences of OMMV variants analysed: WT (OMMV_GP, reference sequence, NC_006939.1), Q20L, Q32R, I133V and A216T.

**Figure 4:**
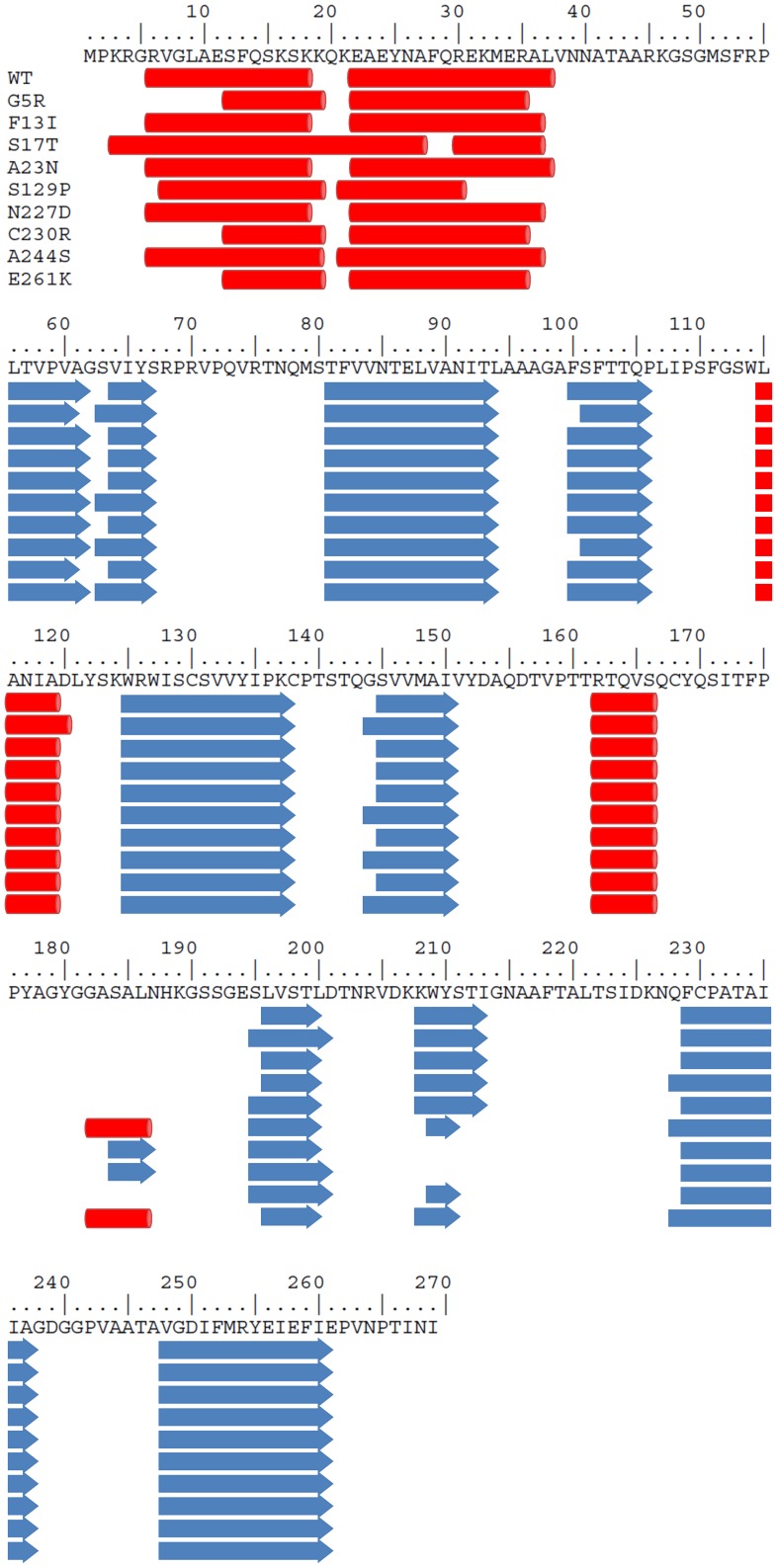
Multiple sequence alignment of the deduced amino acid sequences of TNV-D variants analysed: WT (TNV-D^H^, NC_003487.1), G5R, F13I, S17T, A23N, S129P, N227D, C230R, A244S, E261K.

Analysis with the SIFT server of the OMMV and TNV-D sequence variants has provided a list of substitutions likely to cause structure alterations. These are Q20L, Q32R, I133V, A216T (for OMMV) and G5R, F13I, A23N, S129P, N227D, C230R, A244S, E261K (for TNV-D). Running secondary structure predictions with the JPRED server produced the results shown in Figures 3 and 4. In both cases the results are practically identical, with the substitutions causing little or no effect in the positions and lengths of helices and sheets. The exception is a small length of beta sheet (residues 208–212) which is not predicted when the substitutions N227D and C230R of the TNV-D sequence are not present. The overall result is not surprising, given the high degree of similarity among each sequence group (>85%), only slight changes in structure are to be expected (see below Homology Modelling). Replacements G5R, F13I, A23N (TNV-D) and Q20L, Q32R (OMMV) fall within the putative peptide signal sequence, for which no structural information is available. In this situation predictions are unreliable and no further consideration was given to such substitutions.

### Homology Modelling

**Figure 5:**
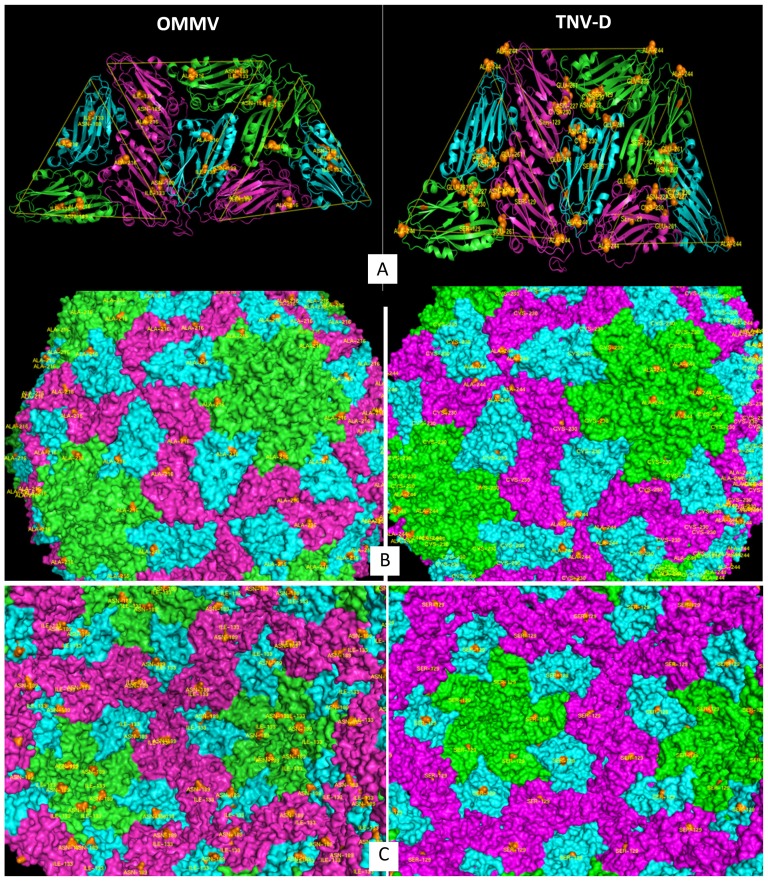
Three-dimensional representation of the OMMV (left) and TNV-D (right) coat protein, highlighting mutations. Mutations are rendered in spheres. Each trimmer is coloured in light green, magenta and cyan. A – Virus minimum contact unit; B – The surface exterior; C – The surface interior.

Given the high degree of identity (≈45%) between the template and target sequences, the virus models were expected, and here confirmed, to be very similar to the template structure. The backbone trace was almost identical in models and experimental structure, with a 0.3 Å RMSD between C-α atoms. The models were based on the crystallographic trimmer (chains A, B and C), and the symmetry operators provided in the PDB entry were used to recreate the entire icosahedral T3 capsid, with a total of 180 chains – this was required to analyze also possible interfacial contacts between monomers. The C chain is 35 residues longer in the models, because its leading sequence is better defined in the X-ray structure than the corresponding sequences of A and B chains. This difference may be related to a specific role of C chains in the assembly of viral capsids. For the analysis of all possible monomer-monomer interactions, a *minimal contact unit* consisting of 3 trimmers was used, where all possible contacts are represented (Figure 5, A).

The models were analysed to investigate the role of the sequence variants that were previously identified as having a potentially disruptive effect on the sequence. As for OMMV sequence variants, the replacement I133V corresponds to the removal of a methyl group from the side chain of I133, creating a small void that may affect interaction with close partner L186. Movement of residue 186 from a C chain could in turn affect interaction with a neighbour C chain. Given the important role of C-chain dimmers in the assembly of this type of virus, this effect cannot be neglected. Residue A216 is in a well exposed location of the outer side of the viral capsid. Replacement by a Threonine residue should have little or no consequence, given the conservative character of the replacement and its unencumbered location. As for TNV-D sequence variants, the mutation S129P, is located on the inner side of the capsid, and not close to any other replaced residue. Proline is a well-known helix breaker, but since the replacement does not occur on a helix no major effects are expected. The Serine residue is polar and hydrogen bond former, while Proline is neither, and that could influence the hydrogen bonding network of residues on the inner side of the capsid. The mutation N227D changes a neutral (but polar) into a negatively charged residue. Given the presence of the nearby (in contact distance) D153, a strong repulsion is expected to arise, that could change the structure of monomer C at its interface with A. Since this replacement is observed together with C230R, another strong attractive interaction will arise between the new Arginine and Aspartate residues at positions 230 and 227. Residue N227 is not in the interior of the capsid, so the replacement should not affect interaction with the viral genetic material. The strong interaction of C230R with residue 227D has been already discussed, but 230R could interact with K226, resulting in a repulsion between these two positively charged residues. (Note: the sequence variants N227D and C230R are the ones where the small segment of β-sheet between residues 208–212 is missing). Mutation A244S results in a change in a residue that is part of the ring structures around the 5-fold and 6-fold axis of the virus capsid. Since there is only a very slight change in size and character, the replacement is not expected to have an impact on the capsid structure. Residue E261 is buried, and lies in contact distance of a group of charged residues, both positive (Lysine, Arginine) and negative (Aspartate, Glutamate). Replacement of the negatively charged Glutamate side chain with a positive Lysine will cause several strong and very close charge-charge interactions that could locally perturb the structure and induce structural changes.

## Discussion and Conclusions

The genetic diversity in OMMV and TNV-D coat protein was evaluated by sequence analysis of the CP of thirteen OMMV isolates and eleven TNV-D isolates, eight of which were obtained from double infected tissues. Additional eight OMMV and three TNV-D CP sequences were retrieved from Genbank and included in this study.

For the amplification of the CP sequences present in the total RNA tissue samples, a single pair of primers common to OMMV and TNV-D was initially used. This resulted in the amplification of TNV-D CP only on the three single TNV-D-infected samples which was confirmed after cloning. In the eight isolates containing mixed infections of OMMV and TNV-D (Table 1), only the presence of OMMV was detected indicating replicase preference for this virus RNA over that of TNV-D. This tallies with previous results that have demonstrated recurrent higher levels of OMMV over TNV-D in olive orchards suggesting that the former is better fitted to the host thus becoming predominant [8]. It is possible that the higher predominance of OMMV is due to a higher virulence when compared to that of TNV-D as observed with other viruses [29]–[32]. Mixed infections, frequent in necroviruses [8], may have important evolutionary implications since they can affect the within isolate population of variants and allow interaction and/or recombination between different viruses thus affecting pathogenicity and adaptability [33]. In fact, it has been proposed that OMMV has originated from genetic recombination between OLV-1 (a virus with which it shares a high identity in the polymerase region) and TNV-D (a virus with which it shares a high identity in the CP region) during their simultaneous replication in the same cell [5].

The analysis of CP gene and of deduced aa sequences revealed that overall diversity of OMMV (average 0.006) and TNV-D (average 0.011) was low either between or within isolates. Diversity values between and within samples were always very near in both virus species isolates, contributing equally to total diversity. This is contrary to results obtained on the alphanecrovirus OLV-1 showing a much higher variability between, rather than within sequences from different OLV-1 isolates [22]. Similar studies on other members of *Tombusviridae* (*Carnation mottle virus* [34], *Pelargonium flower break virus* [21], *Sweet potato chlorotic stunt virus* [35], *Olive latent virus 1* [22]) have shown diversity values for virus CP sequences of near 0.03.

Sequence conservation among the CP of OMMV isolates (nt pairwise distance <0.018 and genetic diversity 0.008) and among TNV-D isolates (nt pairwise distance <0.034 and genetic diversity 0.013) was extremely high in olive isolates. These values were, as expected, higher when sequences of the two viruses were compared (reaching nt pairwise distances and genetic diversities values of 0.411 and 0.199, respectively). However, similar values of pairwise distances and genetic diversities have been observed in other studies among different isolates from a unique virus (*Grapevine leafroll-associated virus* 4) [35]. Based on the deduced amino acid (aa) CP sequences, identities between the several OMMV and TNV-D isolates ranged from 84.3% to 85.8%, as previously observed [3] who found an identity of 85.1% between isolates OMMV_GP and TNV-D^P^.

The comparison among isolates infecting other hosts has shown nt pairwise distances of <0.251 and <0.218 for OMMV and TNV-D, respectively, and genetic diversity of 0.034 and 0.021 for OMMV and TNV-D, respectively. These results seem to indicate some host specificity as already shown in other studies [21], [22], [36], [37].

The ‘S’ domain is the only conserved domain found in OMMV and TNV-D CP sequences, when examined by NCBI conserved domain search software, which may explain the low diversity found since it is the most conserved region in the CP of small plant viruses, indicating that it is where more functional or structural constraints are located [9], [21]. Residues in the ‘S’ domain have shown to be better conserved in all OMMV and TNV-D isolates, whereas the sequences containing the N-terminal region, are slightly less conserved, as previously observed with other TNV strains [13]. The diversity between OMMV and TNV-D in the N-terminal region reaches its higher value of 0.4. Several studies have shown that viruses present lower identity values in this region than in the rest of the CP region [21], [38]. TNV-D, which presents a mean identity of 0.45 in the CP protein with TNV-A has shown a sequence identity of less than 0.2 in this region [13]. Although a high number of basic residues is maintained in this region, residues may have diverged during evolution, probably due to the lack of constraints that act in this N-terminal region, except for the predicted interaction with the RNA inside the particle. The fact that the N-terminal region is buried inside the particle [13] may explain why the surface features of the particle are little affected by this region, and why OMMV and TNV-D presenting a diversity of 40% in this region, show identical serological reactions.

Among the CP sequences compared in detail, 143 residues are completely conserved. Among these are 10 residues previously shown to be conserved among CP sequences of different genera in the *Tombusviridae* [38] and predicted to have an important structural role.

The relatively low genetic diversity found for both OMMV and TNV-D suggests that negative selection restricts the number of molecular variants. This was confirmed after evaluation of selective constraints by comparing rates of synonymous and nonsynonymous substitutions across codon sites. Only one positively-selected codon was detected in OMMV CP and 2 in TNV-D CP, whereas for OMMV, 5 codons were under negative selection, and for TNV-D there were 8, indicating that changes in aa residues would result in functional or structural disadvantages, indicative of a strong negative or purifying selection. All negatively selected sites were, for both viruses, in the ‘S’ domain region, with the exception of 1 codon of OMMV CP gene that was located in the N-terminal region, however it did not correspond to any of the basic residues which are likely to be critical for RNA-binding capability. This has also been observed with other viruses, suggesting that selection is acting on the preservation of the proper conformation of the RNA-binding motif [21], [39].

Negative constraints that viral CPs are subjected to, may be due to multiple functions, including genome encapsidation and protection, cell to cell movement, transmission between plants, host and/or vector interactions, suppression of gene silencing. Chare and Holmes [40] analysed selection pressures in the capsid genes of plant RNA viruses and found that vector borne viruses are subjected to a greater selection than non-vectored viruses. Soil transmission of OMMV is known to be more efficient in the presence of the fungus *Olpidium brassicae* [17], however, a single mutation N189Y, located in the virus particle interior, rendered it non fungus-transmitted probably due to changes in particle conformation affecting the recognition site [14]. Interestingly, this mutation was found in all clones of 7 of the 21 OMMV isolate sequences analysed. A remarkable covariation was found between amino acid positions 189 and 216 of the OMMV CP sequence. Asparagine in position 189 correlated with Alanine in position 216, and the change N189Y correlated to the change A216T, a mutation that is located in the surface of the particle but has however not shown to influence virus transmissibility by the fungus [14]. This covariation suggests the existence of tertiary interactions between these regions of the molecule and that mutation may result from a readjustment of the viral particle towards stability. However, single directed mutants were constructed with each one of these mutations, and the virus remained stable and infectious [14]. As for TNV-D isolates, in place of the mutation A216T, an alanine residue is present (position 213) and in the place of the mutation N189Y, TNV-D isolates show a lysine residue instead of asparagine (position 186). This substitution of a polar aa to other polar aa does not suggest alterations in virus conformation.

Based on the prediction of structure/function of the CP based on the sequences, we found that only the mutations Q20L, Q32R, I133V, A216T in OMMV and G5R, F13I, A23N, S129P, N227D, C230R, A244S and E261K in TNV-D are likely to cause significant alterations in virus structure.

Analysis of the TNV-D and OMMV comparative models shows that replacements A216T of OMMV and C230R and A244S of TNV-D are located in the outer surface of the capsid and replacements I133V of OMMV and S129P of TNV-D are located in the capsid interior (figure 5), while N227D and E261K of TNV-D are buried below the surfaces. The location of the OMMV mutation A216T in the particle surface was predicted previously [14]. OMMV replacement I133V could affect interaction with L186 which in turn could affect interaction with a neighbour C chain, disturbing the capsid assembly and/or changing virion conformation. This may have several implications, namely by changing the accessibility of exposed amino acids in particle surface as was suggested by several authors as a probable reason why interior mutations render loss of vector transmission [14], [41]. Replacement A216T produces a new polar residue at an exposed zone which may have an impact on the interaction of the virus with the host cell. The replacement N189Y has previously been shown to influence virus transmission [14] and it was decided to include this mutation in the analyses even though it was not marked by SIFT as potentially critical. This shows that the preservation of structure does not always correlate with preservation of functions. Residue 189 is placed around the 5- and 6-fold axis, on the inner side of the capsid. They are very well exposed at the tip of a loop thus not very likely to cause steric clashes when replaced by tyrosine. However, this residue is well placed for interaction with the viral genome, and as such their replacement could potentially affect viral properties.

As for TNV-D, replacements N227D and C230R may give rise to strong repulsions due to nearby identically charged residues (D153 and K226) and this could have a significant effect on the capsid assembly and stability, since these residues lie close to the monomer-monomer interfaces. The replacement S129P may affect interaction with the nucleic acid in the capsid interior.

In general, point aminoacid replacements are expected to cause only small effects upon the structure, which could form a strong selective pressure to preserve the coat protein structure. This notion is supported through comparison of the coat proteins of TNV and *Tobacco bushy stunt virus*, with very similar structures (2.5 Å RMSD) but very divergent sequences (14% identity).

All the substitutions under study were naturally occurring variants filtered by natural selection, and not comparable to isolated mutational events many of which would most likely result in large structural changes and malformed or non-assembling viral capsids.

Few studies have been done on the functional role of the CP in OMMV and TNV-D and these focus mainly on the ability of the virus particles to bind to fungal zoospores and act in *in vitro* transmission events [14], [17]. Two single OMMV mutations (N189Y and A216T) did not alter virus infectivity nor systemic movement. However other mutations may act differently at various levels. Thus it would be of interest to find the role of all the predicted important structural mutations found in the CP to investigate the potential implications of these alterations in virus movement, elicitation of symptoms, host range and particle assembly. Some studies concerning the CP of OLV-1 [42] and *Carnation ringspot virus* [43], also of the *Tombusviridae* family, have highlighted the importance of the C-terminal domain on the systemic movement. Among the predicted important structural mutations found, A216T in OMMV and N227D, C230R, A244S and E261K in TNV-D are in the C-terminal region and may have implications at that level.

A possible explanation for the high stability found either in OMMV or TNV-D isolates, sometimes even greater between different isolates than within an isolate, may be that these isolates have evolved from a single original sequence in an original host, possibly olive. The fact that olive material is propagated through cuttings, and that these viruses occur symptomless, contributes to the rapid multiplication and dissemination of the viruses and may lead to a high degree of virus CP similarities. OMMV, as suggested, may have originated from recombination events between OLV-1 and TNV-D. Recombination may play a significant role in the evolution of necrovirus by producing new viruses, rather than point mutations. Several members of *Tombusviridae* have shown to contain putative recombination signals in the genome of some of their members, including TNV-D and OMMV. TNV-D is suggested to result from recombination between BBSV and OMMV isolates and OMMV from recombination between OLV-1 and TNV-D [5], [44]. In the family *Tombusviridae*, recombination in the genera *Carmovirus* and *Tombusvirus* has been shown to repair damaged deleted 3′ends of virus associated RNAs, as well as generate new satRNAs or DIRNAs [45]. Genetic drift may also have contributed to the low variability after bottlenecks virus populations undergo such as systemic movement or transmission between plants. The major changes observed seem to be the result of successful adaptation to other hosts.

Data here presented agree with those of other authors who have reported high genetic stability for several RNA viruses, suggesting the existence of very strong selective pressures for the preservation of their biological functions [19], [46]–[48] and the most variability exists between virus isolates due to host speciation [22]. In addition, it was shown that mere preservation of virus structural identity does not always correlate with functional conservation.

## Supporting Information

Data S1 List of accession numbers of the virus sequences used in this study.
(DOCX)
