# Correction: Genetic Diversity of the Coat Protein of Olive Mild Mosaic Virus (OMMV) and Tobacco Necrosis Virus D (TNV-D) Isolates and Its Structural Implications

**DOI:** 10.1371/journal.pone.0128171

**Published:** 2015-05-08

**Authors:** Carla M. R. Varanda, Marco Machado, Paulo Martel, Gustavo Nolasco, Maria I. E. Clara, Maria R. Félix

The following information is missing from the Funding section: This work is funded by National Funds through FCT—Foundation for Science and Technology under the Strategic Project PEst-OE/AGR/UI0115/2014.

The complete, correct funding statement should read: Carla Marisa R. Varanda is the recipient of a Post doctoral fellowship from Fundação para a Ciência e a Tecnologia (FCT), SFRH/BPD/76194/2011. This work has been supported by FEDER and National funds, through the Programa Operacional Regional do Alentejo (InAlentejo) Operation ALENT-07-0262-FEDER-001871/Laboratório de Biotecnologia Aplicada e Tecnologias Agro-Ambientais. Additionally, this work is funded by National Funds through FCT—Foundation for Science and Technology under the Strategic Project PEst-OE/AGR/UI0115/2014. The funders had no role in study design, data collection and analysis, decision to publish, or preparation of the manuscript.
