# Supplementary material for: Genetic Diversity of the Coat Protein of Olive Mild Mosaic Virus (OMMV) and Tobacco Necrosis Virus D (TNV-D) Isolates and Its Structural Implications
Source: PLoS One. 2014 Oct 28;9(10):e110941. doi: 10.1371/journal.pone.0110941 (PMC4211703; doi:10.1371/journal.pone.0110941)
Supplement: Data S1 — List of accession numbers of the virus sequences used in this study. (DOCX) [file pone.0110941.s001.docx]

Supporting information file: Genbank accession numbers

Varanda et al. “Genetic diversity of the coat protein of Olive mild mosaic virus (OMMV) and Tobacco necrosis virus D (TNV-D) isolates and its structural implications”

Dear GenBank Submitter:

Thank you for your direct submission of sequence data to GenBank. We have

provided GenBank accession numbers for your nucleotide sequences:

BankIt1750695 OMMV_OMMV3-A KM355247

BankIt1750695 OMMV_OMMV3-B KM355248

BankIt1750695 OMMV_OMMVL11 KM355249

BankIt1750695 OMMV_GP-A KM355250

BankIt1750695 OMMV_GP-B KM355251

BankIt1750695 OMMV_GP-C KM355252

BankIt1750695 OMMV_A1P2-A KM355253

BankIt1750695 OMMV_A1P2-B KM355254

BankIt1750695 OMMV_A1P2-C KM355255

BankIt1750695 OMMV_A1P2-D KM355256

BankIt1750695 OMMV_A1P2-E KM355257

BankIt1750695 OMMV_A4P2-A KM355258

BankIt1750695 OMMV_A4P2-B KM355259

BankIt1750695 OMMV_A4P2-C KM355260

BankIt1750695 OMMV_A4P2-D KM355261

BankIt1750695 OMMV_A5P2-A KM355262

BankIt1750695 OMMV_A5P2-B KM355263

BankIt1750695 OMMV_A5P2-C KM355264

BankIt1750695 OMMV_A5P2-D KM355265

BankIt1750695 OMMV_A10P2-A KM355266

BankIt1750695 OMMV_A10P2-B KM355267

BankIt1750695 OMMV_A10P2-C KM355268

BankIt1750695 OMMV_A10P2-D KM355269

BankIt1750695 OMMV_A4P5-A KM355270

BankIt1750695 OMMV_A4P5-B KM355271

BankIt1750695 OMMV_A4P5-C KM355272

BankIt1750695 OMMV_A6P5-A KM355273

BankIt1750695 OMMV_A6P5-B KM355274

BankIt1750695 OMMV_V4 KM355275

BankIt1750695 OMMV_V8-A KM355276

BankIt1750695 OMMV_V8-B KM355277

BankIt1750695 OMMV_V8ia1-A KM355278

BankIt1750695 OMMV_V8ia1-B KM355279

BankIt1750695 OMMV_V10-A KM355280

BankIt1750695 OMMV_V10-B KM355281

BankIt1750695 OMMV_V10-C KM355282

BankIt1750695 OMMV_V10-D KM355283

BankIt1751085 TNVD_V4PB-A KM355284

BankIt1751085 TNVD_V4PB-B KM355285

BankIt1751085 TNVD_V4PB-C KM355286

BankIt1751085 TNVD_V4PB-D KM355287

BankIt1751085 TNVD_V4PB-E KM355288

BankIt1751085 TNVD_V10-A KM355289

BankIt1751085 TNVD_V10-B KM355290

BankIt1751085 TNVD_V10-C KM355291

BankIt1751085 TNVD_V10-D KM355292

BankIt1751085 TNVD_A5P2 KM355293

BankIt1751085 TNVD_TNVD-A KM355294

BankIt1751085 TNVD_TNVD-B KM355295

BankIt1751085 TNVD_TNVD-C KM355296

BankIt1751085 TNVD_TNVD-D KM355297

BankIt1751085 TNVD_A1P2-A KM355298

BankIt1751085 TNVD_A1P2-B KM355299

BankIt1751085 TNVD_A1P2-C KM355300

BankIt1751085 TNVD_A4P2-A KM355301

BankIt1751085 TNVD_A4P2-B KM355302

BankIt1751085 TNVD_A6P5-A KM355303

BankIt1751085 TNVD_A6P5-B KM355304

BankIt1751085 TNVD_A10P2-A KM355305

BankIt1751085 TNVD_A10P2-B KM355306

BankIt1751085 TNVD_GP-A KM355307

BankIt1751085 TNVD_GP-B KM355308

BankIt1751085 TNVD_V6-A KM355309

BankIt1751085 TNVD_V6-B KM355310

BankIt1751085 TNVD_V6-C KM355311

BankIt1751085 TNVD_V6-D KM355312

BankIt1751085 TNVD_V8i KM355313
